# Potential Antileukemia Effect and Structural Analyses of SRPK Inhibition by *N*-(2-(Piperidin-1-yl)-5-(Trifluoromethyl)Phenyl)Isonicotinamide (SRPIN340)

**DOI:** 10.1371/journal.pone.0134882

**Published:** 2015-08-05

**Authors:** Raoni Pais Siqueira, Éverton de Almeida Alves Barbosa, Marcelo Depólo Polêto, Germanna Lima Righetto, Thiago Vargas Seraphim, Rafael Locatelli Salgado, Joana Gasperazzo Ferreira, Marcus Vinícius de Andrade Barros, Leandro Licursi de Oliveira, Angelo Brunelli Albertoni Laranjeira, Márcia Rogéria Almeida, Abelardo Silva Júnior, Juliana Lopes Rangel Fietto, Jörg Kobarg, Eduardo Basílio de Oliveira, Robson Ricardo Teixeira, Júlio César Borges, Jose Andrés Yunes, Gustavo Costa Bressan

**Affiliations:** 1 Departamento de Bioquímica e Biologia Molecular, Universidade Federal de Viçosa, Viçosa, Minas Gerais, Brasil; 2 Instituto de Biologia, Universidade Estadual de Campinas, Campinas, São Paulo, Brasil; 3 Instituto de Química, Universidade de São Paulo, São Carlos, São Paulo, Brasil; 4 Departamento de Química, Universidade Federal de Viçosa, Viçosa, Minas Gerais, Brasil; 5 Departamento de Biologia Geral, Universidade Federal de Viçosa, Viçosa, Minas Gerais, Brasil; 6 Centro Infantil Boldrini, Campinas, São Paulo, Brasil; 7 Departamento de Veterinária, Universidade Federal de Viçosa, Viçosa, Minas Gerais, Brasil; 8 Laboratório Nacional de Biociências, Centro Nacional de Pesquisa em Energia e Materiais, Campinas, São Paulo, Brasil; 9 Departamento de Engenharia de Alimentos, Universidade Federal de Viçosa, Viçosa, Minas Gerais, Brasil; International Centre for Genetic Engineering and Biotechnology, ITALY

## Abstract

Dysregulation of pre-mRNA splicing machinery activity has been related to the biogenesis of several diseases. The serine/arginine-rich protein kinase family (SRPKs) plays a critical role in regulating pre-mRNA splicing events through the extensive phosphorylation of splicing factors from the family of serine/arginine-rich proteins (SR proteins). Previous investigations have described the overexpression of SRPK1 and SRPK2 in leukemia and other cancer types, suggesting that they would be useful targets for developing novel antitumor strategies. Herein, we evaluated the effect of selective pharmacological SRPK inhibition by *N*-(2-(piperidin-1-yl)-5-(trifluoromethyl)phenyl)isonicotinamide (SRPIN340) on the viability of lymphoid and myeloid leukemia cell lines. Along with significant cytotoxic activity, the effect of treatments in regulating the phosphorylation of the SR protein family and in altering the expression of MAP2K1, MAP2K2, VEGF and FAS genes were also assessed. Furthermore, we found that pharmacological inhibition of SRPKs can trigger early and late events of apoptosis. Finally, intrinsic tryptophan fluorescence emission, molecular docking and molecular dynamics were analyzed to gain structural information on the SRPK/SRPIN340 complex. These data suggest that SRPK pharmacological inhibition should be considered as an alternative therapeutic strategy for fighting leukemias. Moreover, the obtained SRPK-ligand interaction data provide useful structural information to guide further medicinal chemistry efforts towards the development of novel drug candidates.

## Introduction

More than 90% of all human transcribed genes may undergo alternative splicing, a mechanism that allows a considerable increase of the encoding potential of eukaryotic genomes [1]. This process leads to the expression of different protein isoforms that may possess even antagonistic functions, as occurs with VEGF pro- and anti-angiogenic isoforms [2,3]. Pre-mRNA splicing flexibility plays a key role in tissue development and cell response to external stimuli, unsurprisingly explaining why dysregulation of these events has been related to the biogenesis of many human diseases, including cancer [2,4].

Regulation of pre-mRNA splicing involves hundreds of auxiliary factors that control splicing site selection, spliceosome assembly and splice reaction [5,6]. The serine/arginine protein family (SR proteins) constitutes an important group of splicing factors responsible for delimitating intron and exon boundaries in pre-mRNAs [5,7]. These proteins act in response to environmental changes and are structurally characterized by an amino-terminal portion possessing one or two RNA-binding domains (RRM) and a carboxy-terminal portion comprising a domain rich in serine and arginine residues (RS) [8,9]. These serine residues are extensively phosphorylated by different kinases, notably by serine/arginine-rich protein kinases (SRPKs) [10]. This kinase family possesses a bi-lobular kinase domain separated by a divergent spacer region that is targeted by molecular chaperones in the context of the EGFR/PI3K/Akt/SRPK signaling pathway [10]. The signaling module EGFR/PI3K/AKT is commonly related to multiple cancers and constitutes the major activation mechanism known for SRPKs to date [11–14]. Activated SRPKs, in turn, regulate SR proteins’ cellular sublocalization, splicing site selection and interaction with additional protein factors [15,16]. Moreover, SRPKs can also activate AKT in the cytosol through a mechanism involving the phosphatase pleckstrin homology (PH) domain leucine-rich repeat protein phosphatase PHLPP [17].

Overexpression and dysregulation of SRPKs have been characterized as important factors in promoting cell proliferation in many human cancers including leukemia, pancreatic, breast, colon, lung, ovarian and melanoma [15,18–22]. Considering leukemias, SRPK1 has been found to be overexpressed in patients with acute lymphoblastic leukemia and chronic myeloid leukemia [23,24]. High levels of SRPK2 in some acute myeloid leukemia cell lines and in acute lymphoblastic leukemia specimens have been correlated to cell proliferation through the acinus SR protein hyperphosphorylation and cyclin A1 expression [15]. In general, it is believed that overexpression of SRPKs may alter the proper activity of SR factors on their primary transcript target, favoring the expression of splicing isoforms that contribute to tumorigenic processes [12]. Hence, the available data suggest that SRPK inhibition could control tumor cell proliferation [15,18].

Previous high throughput screening campaigns have identified the *N*-(2-(piperidin-1-yl)-5-(trifluoromethyl)phenyl)isonicotinamide, also named SRPIN340, as an ATP-competitive inhibitor that is highly selective for SRPK1 and SRPK2 [25,26]. This inhibitor has been characterized as an antiviral agent against the replication of several viruses, such as HIV, Sindbis virus, HCV and hepatitis C [25,27,28]. In addition, it presented anti-angiogenic activity in an ocular murine angiogenesis model [29–32] and antitumor activity when locally injected into human melanoma tumors developed in mice transplanted with human melanoma cells [22].

Considering that SRPK dysregulation can lead to AKT hyperactivation and can additionally generate multiple abnormal protein isoforms involved in the biogenesis and progression of several cancers, this work describes an *in vitro* evaluation of the antileukemia potential of SRPK pharmacological inhibition. In addition, structural data that might explain SRPIN340’s inhibitory activity on SRPK2 are also described.

## Experimental Procedures

### Cell lines

The leukemia cell lines used were K562 (chronic myelogenous leukemia—CML); KG1 and HL60 (acute myelogenous leukemia—AML); Jurkat, TALL, and Molt4 (T-cell acute lymphoblastic leukemia–ALL-T); and RS4, 697, and Nalm6 (B-cell acute lymphoblastic leukemia–ALL-B). K562, HL60, RS4, and 697 were kindly provided by Dr. Sheila A. Shurtleff (St. Jude Children’s Research Hospital, Memphis, TN). The Nalm6 cell line was provided by Dr. Angelo Cardoso (Dana-Farber Cancer Institute, Boston, MA). TALL was kindly provided by Dr. Joao T. Barata (Instituto de Medicina Molecular, Lisboa, Portugal). The KG1, Molt4, and Jurkat cell lines were provided by Dr. Alexandre E. Nowill (Centro Integrado de Pesquisas Oncohematológicas da Infância, UNICAMP, Campinas, Brazil). Cells were cultivated in RPMI 1640 (Sigma) medium supplemented with 10% (v/v) fetal bovine serum (FBS) (LGC Biotecnologia), 100 g/mL streptomycin, and 100 units/mL penicillin at pH 7.2 and 37°C under a 5% CO_2_ atmosphere.

### Isolation of PBMC from human blood

Peripheral blood was collected in EDTA tubes, diluted with an equal volume of Hank’s balanced salt solution (HBSS) and mixed gently. All procedures were performed according to ethics considerations of the Declaration of Helsinki and were approved by the ethics committee of the Universidade Federal de Viçosa. Afterwards, samples were layered onto a cushion of Histopaque 1077 (Sigma) and centrifuged at room temperature for 30 min at 400 x*g*. Mononuclear cells were collected from the interface, washed twice in HBSS, and centrifuged at room temperature for 8 min 240 x*g*. These cells were resuspended in complete RPMI 1640 medium supplemented with 10% fetal bovine serum and 1% (v/v) phytohemagglutinin (Gibco) and counted using a Neubauer chamber for the following experiments.

### SRPK inhibitor synthesis

Compound *N*-(2-(piperidin-1-yl)-5-(trifluoromethyl)phenyl)isonicotinamide (SRPIN340) was synthesized as published previously [26].

### MTT cell viability assay

Leukemic cells (5x10^4^ cells/well) and isolated PBMCs (8x10^4^ cells/well) were seeded in 96-well plates. Each well contained 100 μL of complete RPMI medium and 100 μL of SRPIN340 solution at different concentrations. The compound was diluted in RPMI medium with 10% fetal bovine serum and 0.4% DMSO (v/v). After 48 h of culture, MTT (5 mg/mL, Sigma) was added to the wells (3 h, 37°C). The plates were centrifuged at room temperature for 30 min 500 x*g*, followed by the removal of the MTT solution and the addition of 100 μL/well of DMSO (Sigma) to solubilize the formazan. Absorbance was measured at 540 nm in a microplate reader (Sinergy HT, Biotek). Each experimental procedure was performed in triplicate.

### Flow cytometry assays

Cultured cells were seeded on a 96-well plate at a density of 10^5^ cells/well. After treatments, cells were labeled using an FITC Annexin V apoptosis detection kit I (BD Biosciences) according to the manufacturer’s protocol. Subsequently, cell samples were submitted to analysis by flow cytometry (FACS Verse, BD Bioscience). Results were analyzed by FlowJo software.

### RT-PCR and RT-qPCR Analysis

mRNA was extracted from leukemic cells using Tri Reagent (Sigma) according to the manufacturer’s protocol. Samples were quantified by spectrophotometry (NanoDrop, Thermo Scientific) and analyzed for integrity in 1% agarose gel. Afterwards, the RNA was used for first-strand cDNA synthesis using the Super Script First-Strand kit (Invitrogen) according to the manufacturer’s protocol. Then, the cDNA was used to amplify each fragment of interest by PCR using the GoTaq Green Master Mix (Promega) kit, and the products were separated in 1% or 2% agarose gels. Quantitative gene expression analyses (RT-qPCR) were performed in an ABI Prism 7500 Sequence Detector system (Applied Biosystems) using SYBR Green I dye (SYBR Green PCR Master Mix, Applied Biosystems). cDNAs, obtained as described above, were used as the template for amplifications following the manufacturer's protocols. All primers used in the RT-PCR and RT-qPCR assays are listed in S1 Table.

### Western Blot Analysis

Cells were counted using a Neubauer chamber and washed once in phosphate buffered saline (PBS). Cells were lysed in PBS containing 1% (v/v) NP40, 1 mM EDTA, 150 mM NaCl, protease and phosphatase inhibitors (Sigma), and 10 mM Tris (pH 7.4) at a concentration of 2x10^7^ cells/mL in lysis buffer. Samples were incubated on ice for 10 minutes, lysed by pipetting, and centrifuged for 10 minutes at 15000 x*g* to remove insoluble cellular debris. An equal volume of 2X sample buffer containing 4% (w/v) SDS, 0.2% (w/v) bromophenol blue, 20% (v/v) glycerol, and 100 mM Tris (pH 6.8) was added to the supernatant. Then, the samples were heated to 70°C for 10 min. Approximately 1.5x10^5^ cell equivalents were loaded per well of 10% Bis-Tris SDS–polyacrylamide gel electrophoresis. Afterwards, proteins were transferred to a polyvinylidene difluoride (PVDF) membrane (GE Healthcare), blocked overnight in PBS containing 5% (w/v) skim milk powder, and then incubated for 2 h with primary antibody solutions. Specific kinases were detected using 1:4000 dilutions of anti-SRPK1 and anti-SRPK2 (BD Biosciences). Phosphorylated SR proteins were detected using a 1:1000 dilution of mAb1H4 (Invitrogen) specific for a phospho-epitope common to multiple SR proteins. Each blot was re-probed with a 1:1000 dilution of anti-actin (Sigma), used as an endogenous control in all experiments. Blots were washed in PBS-Tween (PBS-T) and incubated for 2 h in a 1:5000 dilution of a peroxidase-conjugated secondary antibody. Then, proteins were visualized using a Super Signal West Pico Chemiluminescent Substrate Kit (Thermo Scientific).

### Cloning, expression and purification procedures

The clone pCMV-SPORT6-SRPK2 was purchased from the Mammalian Gene Collection (Invitrogen). This clone allowed amplification of full-length SRPK2 cDNA by PCR and subcloning into the pET28a-HIS-TEV vector [33], a modified version of the bacterial expression vector pET28a (Novagen). The following primers were used: forward primer 5'-GAGCTCATGTCAGTTAACTCTGAGAAGTCG-3' and reverse primer 5'-GTCGACCTAAGAATTCAACCAAGGATGCC-3’. Expression of SRPK2 N-terminally fused to 6xHistidine (6xHis) was induced in *Escherichia coli* (BL21) by 0.25 mM isopropyl thio-β-D-galactoside (IPTG) for 2 h at 30°C. After harvesting, the pellets were resuspended in 20 mM phosphate, 500 mM NaCl, and 20 mM imidazole at pH 7.4. Lysis was performed by adding 5 U of DNAse (Fermentas) and 30 μg/mL of lysozyme (Sigma) followed by 30 min of incubation on ice and disruption by 10 cycles of sonication. Supernatants were obtained after centrifugation at 24586 x*g* for 15 min at 4°C. The obtained supernatants were loaded onto a HiTrap Chelating HP column (GE Healthcare) coupled to an AKTA FPLC (GE Healthcare) equilibrated with lysis buffer. The 6xHis-SRPK2 was eluted by a gradient of 0–500 mM. The obtained Ni^2+^ affinity-purified fractions were dialyzed against a buffer containing 10 mM phosphate at pH 7.5. After a 2-fold dilution, samples were then loaded onto a CHT Ceramic Hidroxy Hepatite type II (Biorad) resin ion-exchange column. Proteins were eluted by a gradient of 0–500 mM phosphate. The efficiency of each purification step was verified by 10% SDS-PAGE. The following dialyses were performed against the sample buffer: 25 mM Tris-HCl, 100 mM NaCl, 1 mM β-mercaptoethanol, and 2 mM EDTA at pH 7.5.

### Fluorescence Spectroscopy

Intrinsic tryptophan fluorescence emission was measured using a fluorescence spectrophotometer F-4500 (Hitachi). SRPK2 emission spectra were acquired at 20°C using 1 μM of protein dissolved in 25 mM Tris-HCl (pH 7.5) buffer, 100 mM NaCl, 1 mM EDTA and 1 mM β-mercaptoethanol in a 1.0 x 0.2 cm quartz cuvette. Tryptophan residues were excited at 295 nm, and the fluorescence emission was collected from 300 nm to 420 nm. SRPK2 spectra were analyzed by means of the maximum fluorescence wavelength (λ_max_) and the spectral center of mass (<λ>) following the equation <λ> = (**Ʃ**λ_i_F_i_)/(**Ʃ**F_i_), where λ_i_ is the fluorescence wavelength and F_i_ is the fluorescence intensity at a given wavelength.

SRPK2 at 1 μM was titrated with SRPIN340 or the ATP analog adenosine 5'-(beta, gamma-imino)triphosphate (AMPPNP). All spectra were corrected by inner filter effects due to ligand absorption, and the dissociation constant (K_D_) for the SRPK2-SRPIN340 interaction was calculated as described previously [34].

### Computational Analyses

Atomic charges and optimal geometry of the tridimensional structure of SRPIN340 were calculated by DFT quantum calculations at the B3LYP/6-31G* level using the CHELPG method [35]. All calculations were carried out in GAMESS software [36]. Then, quantum charges were implemented to the topology generated by the PRODRG server [37].

All SRPK2 structures found in the Protein Data Bank (PDB; www.pdb.org) repository are truncated forms lacking the spacer insert domain (SID), amino acids A236-R507. Structure PDB ID 2X7G was solved based on a SRPK2 recombinant version presenting the segments A236-P256 and A508-D510, generating a SID-like loop. Because this 2X7G crystal structure lacks some coordinates of SID-like loop atoms, they were modeled using the Swiss Model server. The obtained model SRPK2-SID-like loop comprising the amino acids A236-P256 and A508-D510 was then used as a target for SRPIN340 docking. For simplification, "SRPK2-SID-like loop structure" is herein named "SRPK2 structure". Docking assays were carried out inside of the SRPK2 ATP binding pocket, as previously identified for SRPK1 [38]. Fifty poses of SRPIN340 bound to SRPK2 were generated using the software Auto Dock 4.2.5.1, exploring the conformational space of the ligand through a genetic algorithm while the target protein was considered a rigid body [39]. Poses were ranked according to the lowest energy and best interaction network using the Auto Dock 4 scoring function [40].

Molecular dynamics simulations were carried out on the SRPIN340/SRPK2 complexes using the GROMACS 4.5.5 software package [41]. First, SRPK2 topology was built using the Gromos53a6 force field [42]. A single point charge extended (SPC/E) water model was used to fill up a cubic water box built around previously generated SRPIN340/SPRK2 complexes. Moreover, amounts of Na^+^ and Cl^-^ corresponding to a concentration of 0.15 M were added to the system to neutralize protein net charges and mimic the cellular environment. After a careful minimization protocol, equilibration protocols were applied to ensure a proper thermodynamic description of the system. The system was equilibrated for 4 ns using *NVT assemble* (constant number of moles, volume and temperature) and *NPT assemble* (constant number of moles, pressure and temperature), consecutively. Additionally, restraint forces (Fc = 1000 kcal/mol.nm²) were used for all non-hydrogen atoms to reach a temperature stabilization of 298 K and pressure stabilization of 1 bar. Afterwards, a non-restrained production phase of molecular dynamics was performed using an integration step of 2 fs by the leap-frog algorithm for a trajectory of 400 ns. A cut-off of 10 Å was adopted to calculate long-range interactions. Simulations were performed in a cluster *Silicon Graphics International* (SGI Inc.).

### Statistical analysis

All numeric data were derived from at least three independent experiments and are shown as means ± standard deviation. Analyses were performed using Microsoft Excel (Microsoft Office Software), GraphPad Prism (GraphPad Software Inc.) and ImageJ. Statistical analyses were done by paired Student’s T tests. *P <0.05 or **P <0.01 was considered significant.

## Results

### Expression of SRPK1 and SRPK2 in leukemia cells

To expand previous findings [15] and gain further information about the relative proportion between SRPK1 and SRPK2 expression among the leukemia cells used in this work, we evaluated the expression of SRPK1 and SRPK2 in a panel of leukemia cell lineages from different origins and genetic backgrounds.

**Fig 1:**
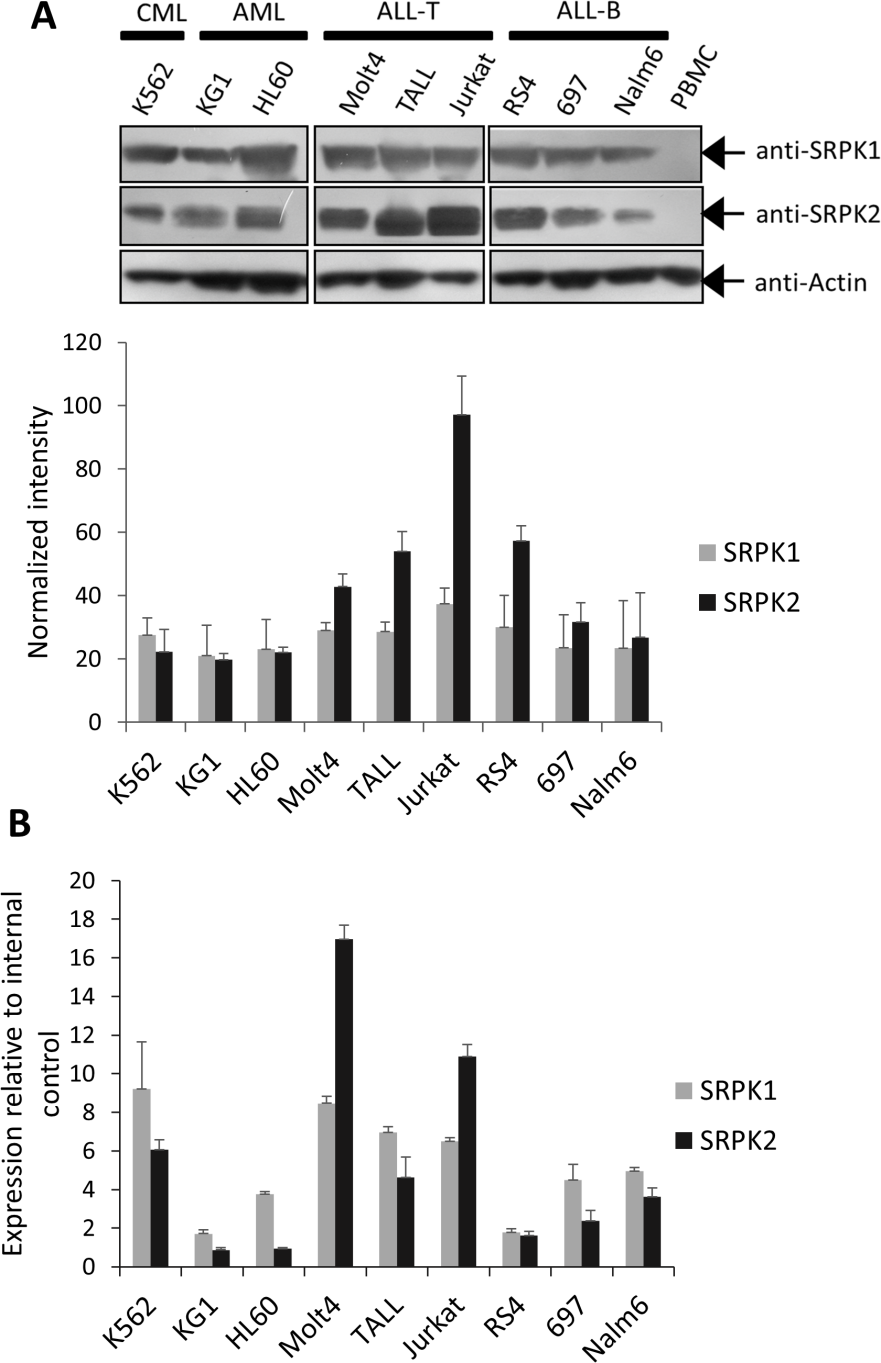
Analysis of SRPK1 and SRPK2 expression in leukemia cell lines. The expression of SRPK1 and SRPK2 were analyzed by (A) Western blotting and (B) RT-qPCR assays in different leukemia cell lines derived from chronic myelogenous leukemia (CML), acute myelogenous leukemia (AML), T-cell acute lymphoblastic leukemia (ALL-T), and B-cell acute lymphoblastic leukemia (ALL-B). (A) The histogram (below) represents the ratio of the band intensities of SRPK1 and SRPK2 normalized to the actin signal for each lineage. Densitometry analysis of the band intensity was performed using ImageJ software. Error bars represent means ± standard deviation from triplicate experiments. Because SRPK1 or SRPK2 signals in the PBMC samples could not be detected during the WB assays, even when higher amounts of material were used (data not shown), they were not considered for the densitometry analysis. Although we found that actin expression varied between leukemia cells and our PBMC samples (S1 Fig), actin was detected here to qualitatively control the presence of protein material. (B) Expression of SRPK1 and SRPK2 transcripts by relative quantification. Amplification of beta-2-microglobulin mRNA (B2M) was used as an endogenous control. B2M was equally expressed among all of the leukemia lineages evaluated (S1 Fig and data not shown). SRPK1 and SRPK2 mRNA quantification in PBMC are discussed in S1 Fig. All primers used are detailed in S1 Table.

Whereas no large differences in SRPK1 protein expression among the lineages were observed, at the protein level (Fig 1A), SRPK2 presented a distinct elevated expression in lineages of lymphoid origin, such as Molt4, TALL, Jurkat (ALL-T) and RS4 (ALL-B). Additionally, Molt4, TALL, Jurkat and RS4 cells presented a higher SRPK2 protein expression compared to SRPK1. Further RT-qPCR experiments revealed that among the nine lineages studied, only Molt4 and Jurkat presented correlations between mRNA and protein levels, indicating that additional layers of complexity involving the gene expression control of these kinases might exist in these cells, which deserves additional investigation in future studies (Fig 1B). We also used human PBMC as a control for non-transformed cells in Western blotting and RT-qPCR experiments. However, SRPK1 and SRPK2 were barely expressed (S1 Fig) or were even undetected in these assays (Fig 1A).

Taken together, these results demonstrate that the expression of SRPK1 and SRPK2 are proportionally distinct among the leukemia cells analyzed. Moreover, a clear correlation between mRNA and protein levels could not be found, reflecting the genetic diversity of these cells and possibly their outcomes during drug treatments.

### Effect of SRPK pharmacological inhibition on leukemia cell viability and death

The emerging relevance of SRPKs as targets for pharmacological intervention has motivated the identification of the inhibitor SRPIN340 (SR Protein Inhibitor 340) and, more recently, its derivative SPHINX (SR Protein Inhibitor X). These compounds seem to possess equivalent biological properties in terms of potency, inhibition of SR protein phosphorylation, reduction in the expression of pro-angiogenic VEGF, and suppression of choroidal neovascularization [43]. Because SRPIN340 activity has been extensively characterized and used in different studies to date, it was chosen as a prototype for pharmacological intervention in leukemia cells here.

**Fig 2:**
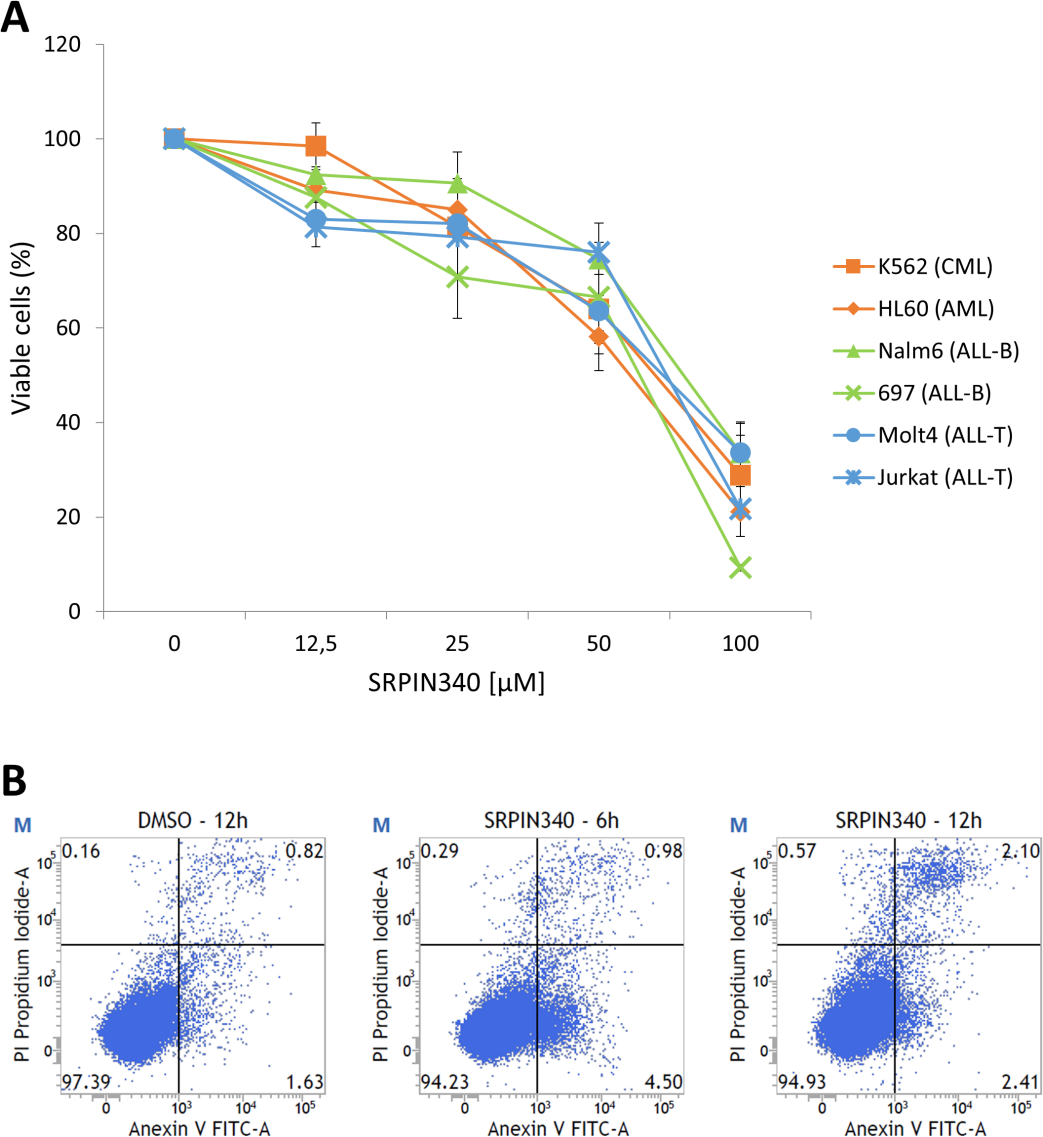
The effect of SRPIN340 treatment on leukemia cell viability and death. Different leukemia cell lines (A) were treated with increasing concentrations (0–100 μM) of SRPIN340 for 48 h. Cell viability was determined using the MTT assay. We considered the viability of 100% of the cells in the control treatment (vehicle). The percentage of inhibition was calculated relative to cells treated with the vehicle. The values are expressed as the means ± standard deviation of three independent experiments. To assess cell death (B), Jurkat cells were treated with 25 μM of SRPIN340 for 6 or 12 h. Cells treated with the vehicle were used as controls. Subsequently, the cell death was evaluated using annexin V-FITC (V) and PI (P) labels. One representative experiment of three is shown.

The cytotoxic potential of SRPIN340 was evaluated against CML, AML, ALL-B and ALL-T cell lineages. All evaluated cell lines were sensitive to the treatments, indicating that SRPK inhibition indeed yields an overall antileukemia effect *in vitro* (Fig 2A). Half-maximal inhibitory concentration values (IC_50_) (Table 1) were determined and revealed that, in general, myeloid leukemias were more sensitive than lymphoid ones. In this case, the AML HL60 was the most sensitive (IC_50_ of 44.7 μM) compared with ALL-T Molt4 and Jurkat (IC_50_ values of 92.2 μM and 82.3 μM, respectively). Moreover, although an accurate IC_50_ value could not be determined at higher compound concentrations, PBMC seemed to be less sensitive to SRPIN340 than the leukemia lineages evaluated. This suggests that SRPK pharmacological inhibition can selectively affect tumor cell survival.

**Table 1:** Half-maximal inhibitory concentration (IC_50_) values for SRPIN340 treatments. Different leukemia cell lines and peripheral blood mononuclear cells (PBMC) were treated with increasing concentrations (0–200 μM) of SRPIN340 for 48 h. Cell viability was determined using the MTT assay. The values are expressed as the means ± standard deviation of three independent experiments.

Cell line	SRPIN340 IC_50_ [μM]
K562 (CML)	52.0 ± 0.6
HL60 (AML)	44.7 ± 2.4
Nalm6 (ALL-B)	66.6 ± 2.7
697 (ALL-B)	59.2 ± 0.6
Molt4 (ALL-T)	92.2 ± 15.2
Jurkat (ALL-T)	82.3 ± 1.2
PBMC	>100*

* The precise IC_50_ value could not be determined due to the low solubility of SRPIN340 at higher concentrations.

Furthermore, Annexin V/PI staining assays were performed to evaluate whether the effect of SRPIN340 treatment impacts Jurkat apoptosis. After 6 h of drug exposure, the cell population in the initial events of apoptosis (Annexin V positive) was approximately twice as high as the control group treated with DMSO for 12 h (values ranged from 1.6 to 4.5%) (Fig 2B). Increasing exposure time to the compound resulted in raising the PI/Annexin V positive cells, indicating the occurrence of later events of apoptosis (Fig 2B). Therefore, SRPIN340 can trigger early and later events of apoptosis in leukemia cells.

### Impact on SRPK cellular activity

**Fig 3:**
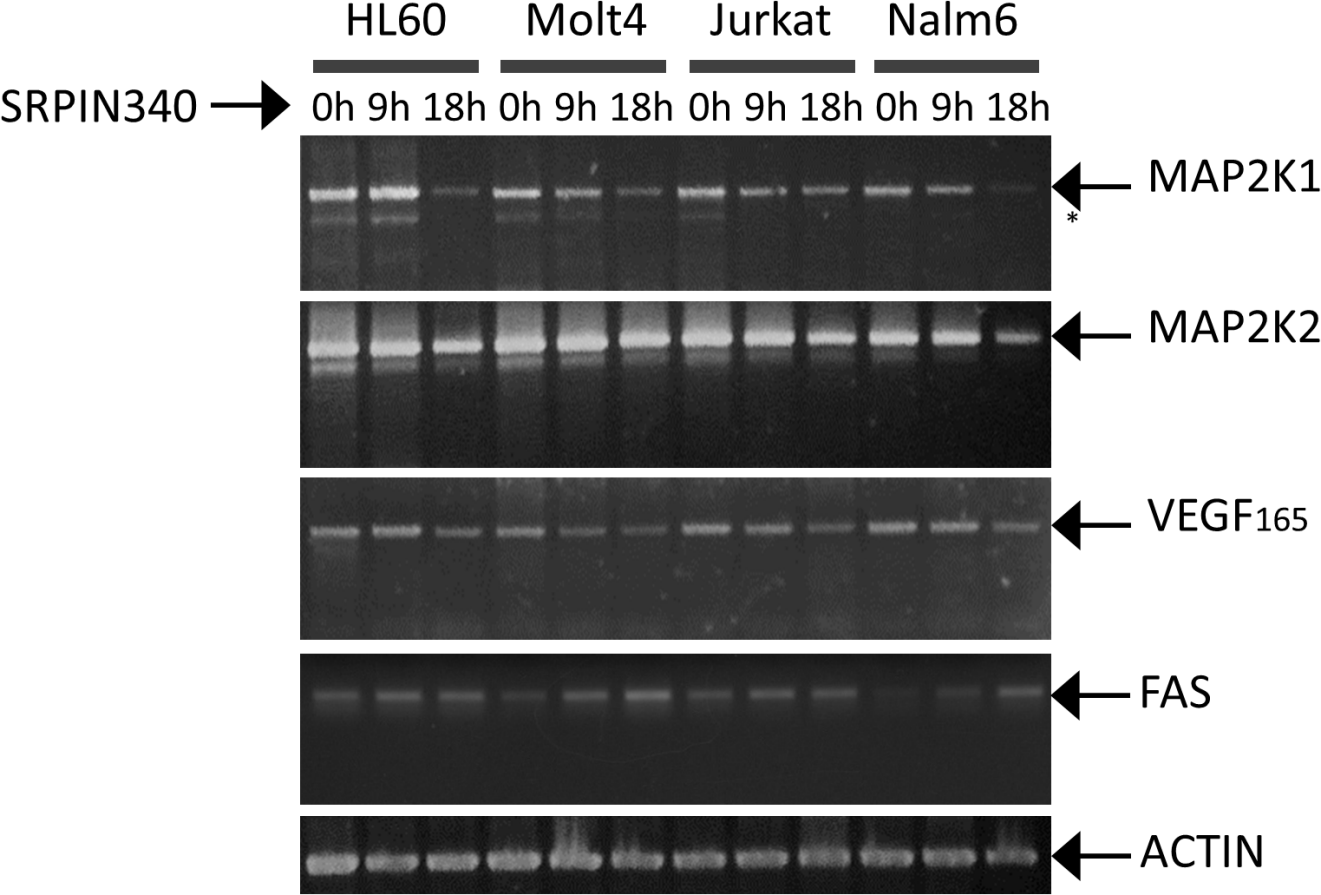
Effect of SRPIN340 treatment on MAP2K1, MAP2K2, VEGF and FAS expression in leukemia cells. cDNAs were derived from HL60, Jurkat, Molt4 and Nalm6 cells treated with SRPIN340 (100 μM) for different amounts of time (0, 9 and 18 h). One representative experiment of three is shown. (*) represent possible isoforms as previously described [19].

In the following experiments, we attempted to confirm whether SRPIN340 treatment affects cellular pathways targeted by SRPKs. As was previously reported, the expression or splicing pattern of transcripts encoding for MAP2K1, MAP2K2, VEGF and FAS are influenced by SR proteins or SRPK activity [19,32,44]. We observed that both MAP2K transcripts had their expression impaired during SRPIN340 treatment (Fig 3). These effects were more pronounced for MAP2K1, which had its expression reduced mainly at a prolonged incubation time. Additionally, we observed a reduction in the pro-angiogenic VEGF_165_ isoform and an increase in the pro-apoptotic FAS isoform expression during the treatments. Although we could not detect clear changes in splicing in leukemias, SRPIN340 changed the splicing of MAP2K1 in HeLa cells (S2 Fig), suggesting that different cancer lineages may respond differently to SRPK inhibition.

**Fig 4:**
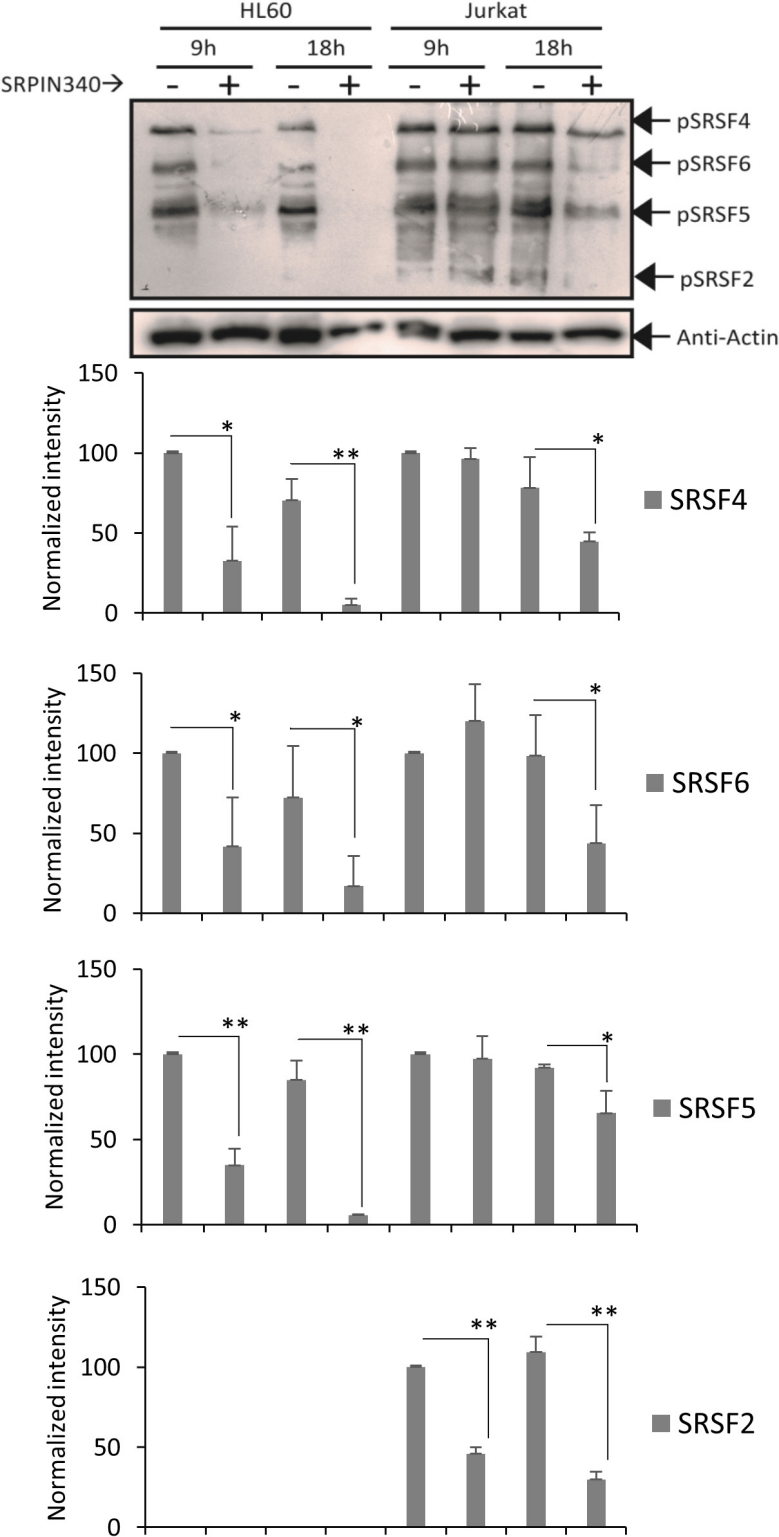
Effect of SRPIN340 treatment on SR protein phosphorylation. Western blotting analysis after treatment with SRPIN340 (100 μM) or negative control (vehicle) for 9 or 18 h. SR protein phosphorylation was detected using mAb1H4, which recognizes phosphorylated serine-arginine epitopes common to multiple SR factors. The blot was re-probed with actin and used as an endogenous control. Graphics (below) represent the percentage of the SR proteins’ band intensity normalized to the actin signal for each HL60 and Jurkat negative control. Densitometry analysis was performed using ImageJ software. Error bars represent the means ± standard deviation from triplicate experiments. T tests, *P < 0.05, **P < 0.01.

The SR protein phosphorylation status was also investigated using a monoclonal antibody able to detect different phospho-SR protein epitopes [45,46]. As expected, the SR protein phospho-epitope signal decreased during treatments of both the HL60 and Jurkat lineages (Fig 4).

Overall, these results confirm that the observed decrease in leukemia cell viability upon SRPIN340 treatment is related to an effect on pathways targeted by SRPK activity.

### Insights on SRPIN340-SRPK complexes

**Fig 5:**
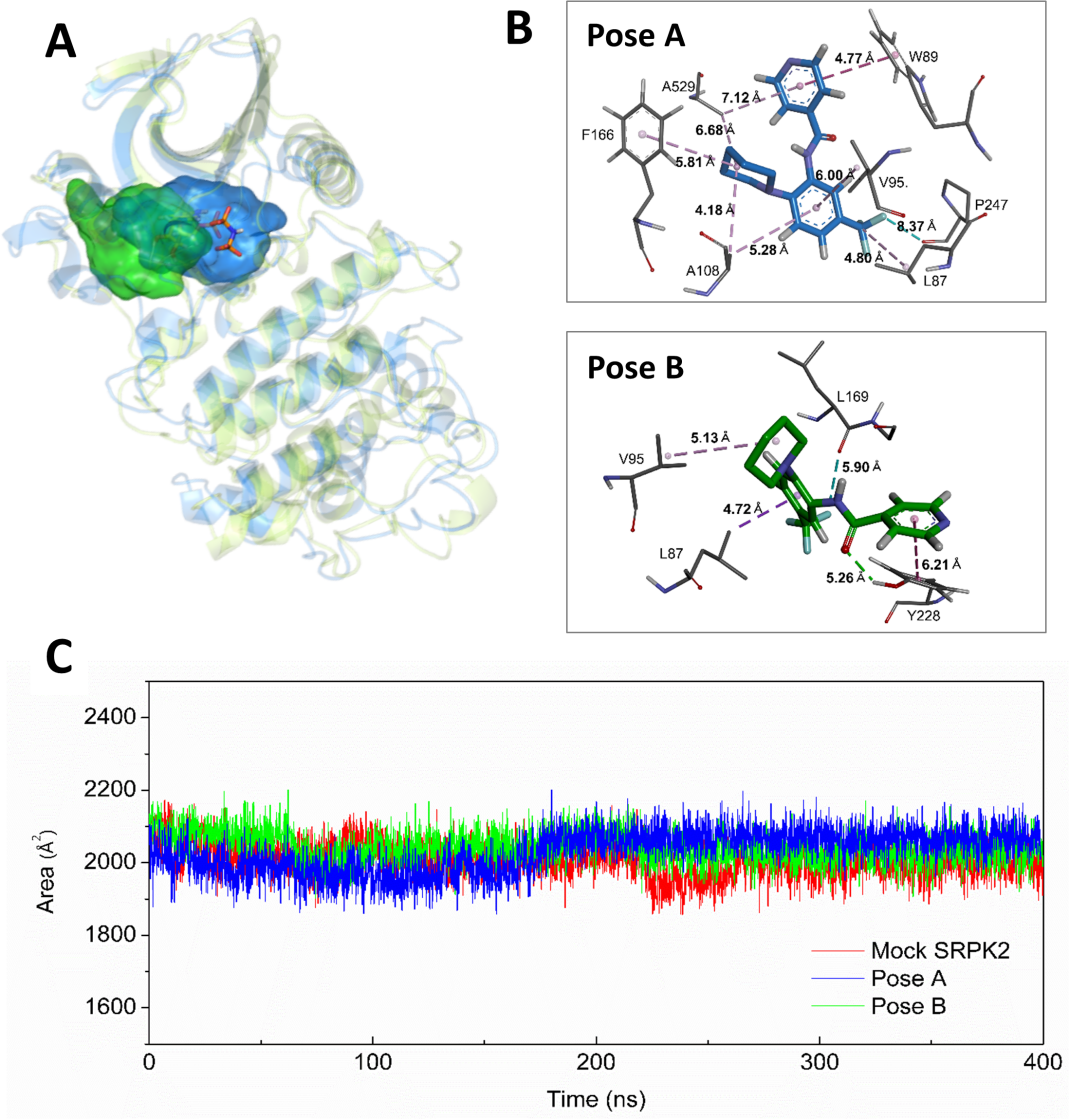
Molecular Dynamic Analysis. (A) Occupied space of SRPIN340 during simulations from poses A (blue) and B (green). Average protein structures are shown superposed in light blue for simulation A and in light green for simulation B. The ATP analogue ANP (shown in orange sticks) occupies the same region as SRPIN340 during both simulations. (B) Snapshot from pose A (upper) and pose B (bottom) simulations highlighting the possible SRPIN340 (orange sticks) interaction network. (C) Solvent accessible surface area (SASA) for tryptophan residues in the presence of the ligand, blue and green for poses A and B, respectively. The pose A simulation shows a signal increase at 175 ns, indicating a major exposure of residues to the polar solvent from this point of simulation. This is in agreement with the fluorescence spectroscopy analysis shown in Fig 6.

Once we observed the pharmacological effect of SRPIN340 in leukemia cells, we sought to investigate possible molecular interactions underlying its binding to SRPKs. While high-resolution crystallographic structures of the complex SRPIN340/SRPKs have not been solved to date, computational approaches were used to infer possible binding sites into SRPK structures currently available in PDB. A comparison of amino acid sequences revealed that both kinases are highly similar in their kinase domains [47] (S3 Fig), explaining why SRPIN340 can inhibit both SRPK1 and SRPK2 [25]. Considering this structural similarity, the position of the ATP analog, previously co-crystallized with SRPK1 [38], allowed the prediction of its binding site into SRPK2 (Fig 5A), the paralog chosen to be studied herein, because its mechanisms in leukemia tumorigenesis have been previously well determined [15].

Because SRPIN340 behaves as an ATP competitive inhibitor [25], docking assays were carried out inside the ATP binding pocket. To take into account protein flexibility and eventual induced fit effects, SRPIN340/SRPK2 complexes corresponding to the best two poses obtained by docking procedures, namely "pose A" and "pose B", were selected and used as inputs for molecular dynamics simulations. RMSD calculations demonstrated that both systems reached stability after 200 ns of simulation (data not shown). In both complexes, the SRPIN340 molecules displayed stable conformations inside the SRPK2 catalytic pocket after 200 ns, maintaining the same network interactions throughout the additional 200 ns. The space occupied by SRPIN340 on pose A and B throughout simulation is shown as a blue and green area, respectively (Fig 5A).

Interestingly, the ATP analog docked into ATP binding site (orange sticks) clearly shows higher superposition to the blue region occupied by SRPIN340 during the molecular dynamics of "pose A", which then seems to better explain the mechanism of competitive inhibition. In this sense, SRPIN340 at "pose A" is additionally stabilized by T-shaped and sandwich pi-stacking interactions with W89 during the last 200 ns (Fig 5B). The simulations also show residues L87, W89, V95, A108, F166, L169, Y228, A529 and P247 acting together to form a network of hydrophobic interactions with SRPIN340. These contacts on the SRPK2/SRPIN340 complex were measured throughout the trajectory in the last 200 ns (Table 2). The most prominent biding groups, with low distance standard deviations, were a benzene ring, CF_3_ and a pyridine ring, either in pose A or B.

**Table 2:** Average distances of SRPIN340 groups and SRPK2 binding site amino acid side-chains. Distances from the SRPIN340 groups and the amino acid side-chains’ centers of mass were measured throughout both simulations. Standard deviation values were considered good for SD < 0.5 Å, medium for 0.5 Å < SD < 1.0 Å and long for SD > 1.0 Å.

**Pose A**		
	**Distance Average (Å)**	**Standard Deviation (Å)**
A108: Benzenic ring	5.28	0.39
L87: CF_3_	4.80	0.45
W89: Pyridine ring	4.78	0.48
A108: Piperidine ring	4.18	0.53
F166: Piperidine ring	5.81	0.80
L87: Benzenic ring	5.66	0.57
V95: Benzenic ring	6.00	0.62
A529: Piperidine ring	6.68	1.28
A529: Pyridine ring	7.12	1.54
P247: CF_3_	8.38	3.93
**Pose B**		
	**Distance Average (Å)**	**Standard Deviation (Å)**
L87: Benzenic ring	4.72	0.45
V95: Piperidine ring	5.13	0.38
Y228: N-cyclohexane	6.21	0.69
L169: CF_3_	5.90	1.63
Y228: O-amide	5.26	1.77

Furthermore, a solvent accessible surface area (SASA) analysis revealed more exposure to polar solvents of tryptophan amino acid residues in structures of "pose A" by approximately 100 Å^2^ (Fig 5C). This finding might have interesting implications if considered together with the following intrinsic tryptophan fluorescence analysis confirmation (see below).

Together, these data can yield important information for the rational design of novel derivatives with increased potency and pharmacological potential.

### Intrinsic tryptophan fluorescence studies

**Fig 6:**
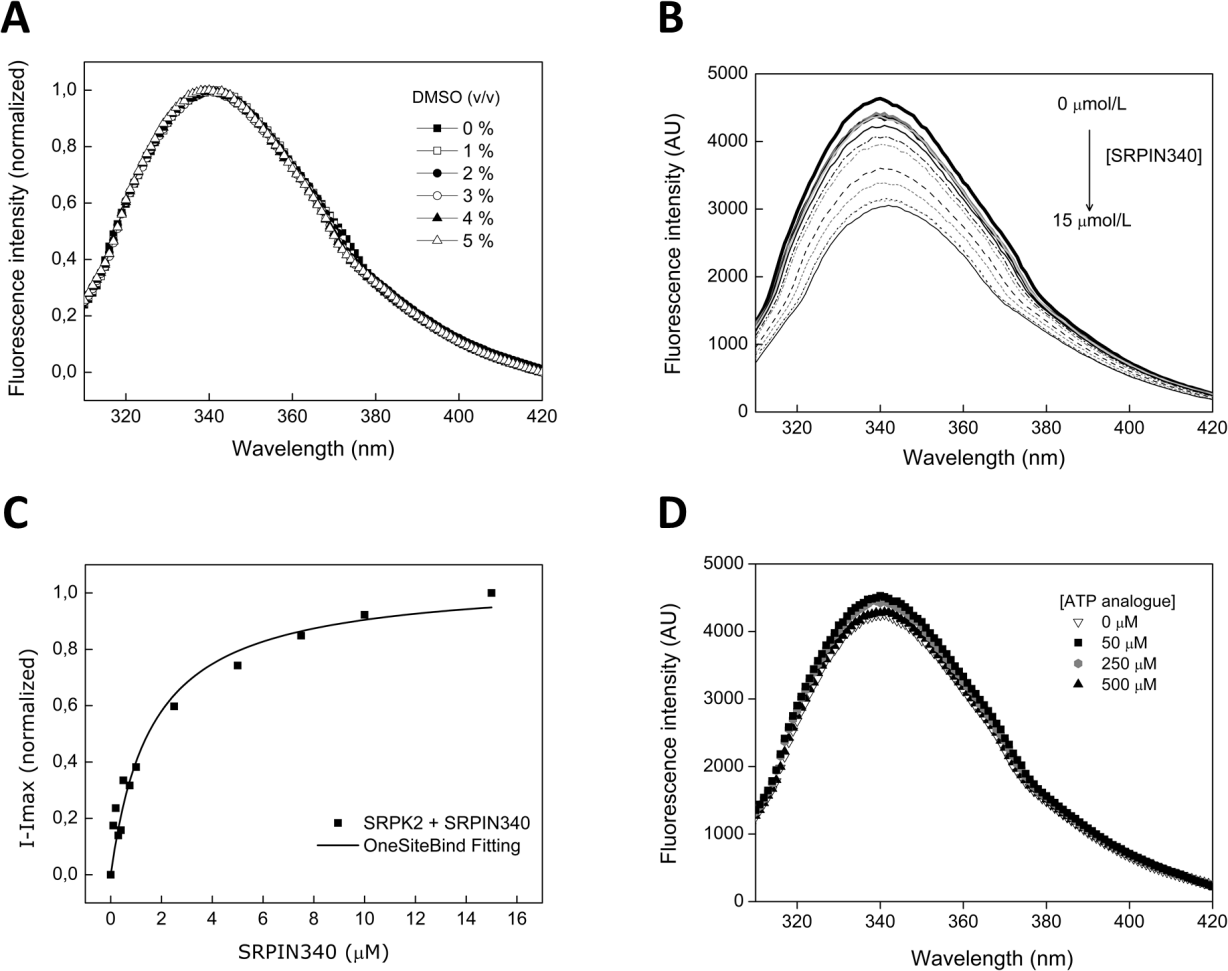
SRPK2/SRPIN340 interaction studied by fluorescence spectroscopy. (A) DMSO titration (0–5% v/v) of SRPK2 spectra showing no significant changes in the local tertiary structure of the W residues. (B) Fluorescence spectra of the SRPK2 titration with increasing concentrations (0–15 μM) of SRPIN340. (C) Data analysis of the spectra shown in (B) and SPRK2/SRPIN340 dissociation constant (K_D_) determination. The K_D_ found for this interaction was 1.6 ± 0.3 μM. (D) SRPK2 (1 μM) fluorescence spectra upon addition of several concentrations of ATP analogue.

To further understand the structural aspects of SRPK inhibition by SRPIN340, fluorescence spectroscopy experiments using tryptophan residues as probes were carried out. The SRPK2 fluorescence spectrum at a λ_max_ of 340 ± 1 nm and a <λ> of 347.1 ± 0.6 nm, indicates that, on average, the SRPK2 W residues are partially exposed to the polar solvent (Fig 6A). For a control, the effect of DMSO (vehicle) on the tertiary structure was also evaluated. Concentrations up to 5% of DMSO did not affect the overall SRPK2 W emission spectrum, assuring that all observed effects are due to the presence of SRPIN340 in solution (Fig 6A).

The titration of SRPK2 with SRPIN340 resulted in a clear intrinsic fluorescence suppression (Fig 6B), as well as in a slight redshift in the λ_max_ and <λ> equal to 342 ± 1.0 nm and 351 ± 0.6 nm, respectively, at 15 μM SRPIN340 (Fig 6B). These results indicate an increase in W residues exposed to the solvent, suggesting that SRPK2 undergoes conformational changes in the presence of SRPIN340 (see also Fig 5C). The titration data additionally allowed the calculation of the dissociation constant (K_D_) for the SRPK2/SRPIN340 interaction equal to 1.6 ± 0.3 μM (Fig 6C). Furthermore, tryptophan fluorescence analysis upon ATP analogue addition revealed no significant effects on the SRPK2 structure, even at higher ligand concentrations (Fig 6D). These data suggest that, compared to ATP, SRPIN340 may undergo different binding modes or access a different interaction network at the SRPK ATP binding pocket, but still act as its inhibitor, which agrees with the computational analyses performed (Fig 5).

## Discussion

Dysregulation of SR protein phosphorylation by SRPKs has been shown to play a key role in cancer [12]. In leukemia cells, SRPK2 overexpression leads to hyperphosphorylation of the splicing factor acinus, which in turn is responsible for increasing the transcription level of cyclin A1 and affecting cell proliferation [15]. SRPK1 and SRPK2 have also been observed to be overexpressed in various other human cancers, including pancreatic, breast, colon, lung and ovarian [18–20,48]. SRPK-altered expression impacts the overall pre-mRNA normal splicing and gene expression program, allowing cancer cells to increase their proliferation, invasiveness, angiogenic potential and apoptosis escape [15,49]. Moreover, high SRPK levels have also been co-related to tumor grade and resistance to chemotherapy [18,19,48]. Thus, much accumulated data points to the fact that SRPKs are functionally relevant for tumor cells, revealing that they can serve as a target for pharmacological intervention.

In previous immunoblotting analyses, high levels of SRPK2 have been found in leukemic bone marrows samples (n = 5), but SRPK2 is undetectable in normal bone marrow counterparts [15]. To find additional information on SRPK expression in leukemia, we analyzed the protein and mRNA levels of SRPK1 and SRPK2 in different leukemia cell lineages herein (Fig 1). Both kinases were found with elevated expression in all cell lines evaluated, but none of them could be detected at significant levels in the PBMCs by Western blotting or RT-qPCR (Fig 1 and S1 Fig). These data are in agreement with previous studies reporting that SRPK1 and SRPK2 are overexpressed in primary bone marrow leukemia samples in comparison with healthy ones [15,23,24].

Additionally, considering protein levels, the proportions of SRPK1 and SRPK2 were different among the lineages studied, reflecting the existence of different regulatory mechanisms operating in these cells to yield altered SRPK1 and SRPK2 expression. It is important to notice that the SRPK2 protein expression was higher than that of SRPK1 in the lineages ALL-T (Molt4, TALL and Jurkat) and ALL-B (RS4) (Fig 1A). This difference is intriguing and certainly would impact therapies based on compounds presenting specificity for SRPK1 or SRPK2. Therefore, these gene expression data, along with others previously reported [15,23,24], reinforce the fact that the therapeutic potential of SRPK inhibition should be considered against hematological malignances.

Based on these accumulated SRPK1 and SRPK2 gene expression data, we aimed to investigate the cytotoxic effect of the SRPK inhibitor SRPIN340 on leukemia cells [25,26]. This substance has been previously shown to be selective for SRPK1 and SRPK2 because no relevant inhibitory activity was observed against a panel of more than 140 kinases, including Cdc-like kinase family members (Clks) that are also involved in SR protein phosphorylation and regulation [25]. Although SRPIN340 was first described as an antiviral agent [25,27,28], its pharmacological activity has also been evaluated in other studies. For instance, it was able to prevent neovascularization in a rodent model of choroidal retinopathy of prematurity [30]. In addition, this compound exhibits antiangiogenic and anti-melanoma effects both *in vitro* and *in vivo* [22]. In the present investigation, SRPIN340 was shown to reduce cell viability of myeloid and lymphoid leukemias *in vitro* (Fig 2 and Table 1). This effect seemed to be selective, as lower cytotoxicity was observed in PBMC treatments. This finding is in agreement with the low apparent toxic effect observed when SRPIN340 was evaluated in *in vivo* studies [25,26].

Furthermore, Annexin V staining cell death assays showed that SRPIN340 cytotoxicity involves the triggering of early and late apoptosis, corroborating previous studies that have shown increased tumor cell sensitivity to cisplatin and gemcitabine during SRPK knockdown experiments [15,18]. Interestingly, the Jurkat and Molt4 lineages seemed more resistant to SRPIN340 treatment (Table 1). This finding might be due to the comparatively higher SRPK2 protein expression in these cells (Fig 1A). It is well known that SRPIN340 has a higher inhibitory activity over SRPK1 compared to SRPK2 [50], implying that this compound would be less effective against tumor cells with higher SRPK2 expression.

Because the SR protein phosphorylation status was reduced during treatments, the SRPIN340 effect on leukemia cells seemed to be associated with SRPK inhibition (Fig 4). It is also interesting to notice that the effect on SR protein phosphorylation in HL60 seemed to be more effective than in Jurkat, which might be again explained by the differences in the proportion of SRPK1/SRPK2 in these cells.

Additionally, we observed in RT-PCR assays that SRPIN340 treatment impaired the expression of MAP2K1 and MAP2K2, both responsible for activating the MAPK3 and MAPK1 pathways [19]. The treatments led to a reduced expression principally of MAP2K1, in which decreased levels were also accompanied by a decreased expression of possible smaller variants (Fig 3). In contrast, when we treated HeLa cells with SRPIN340, a smaller splicing variant of MAP2K1 was expressed, indicating that the compound can favor spliced isoforms with less tumorigenic potential (S2 Fig). However, the same effect on splicing could not be observed for MAP2K2, VEGF or FAS in leukemia or HeLa cells, suggesting that different tumor lineages may respond differently to SRPK inhibition. When similar analyses were performed with breast, colonic, and pancreatic carcinoma lineages, the SRPK1 knockdown impacted the expression of MAP2K2 instead of MAP2K1 [19], providing additional evidence for this different response according to each cell line. Regardless, the impaired gene expression observed in our assays indicates that cell survival pathways involving MAPK3, MAPK1 and Akt are affected by SRPIN340 treatment.

SRPIN340 reduced the expression of pro-angiogenic isoform VEGF_165_ in all leukemia cell lines evaluated, mainly after 18 h of treatment. At the same time, pro-apoptotic FAS expression was induced (Fig 3). These data give more support, as previously described, that SRPK inhibition may be related to triggering apoptosis [19,51]. Additionally, recent studies have shown that SRPK1 targeting is a reasonable strategy for cancer treatment due to the inhibition of angiogenesis [22,32]. Thus, the key role played by SRPKs in regulating cellular death or proliferation offers novel opportunities to develop antileukemia therapies.

The *in vivo* anti-melanoma effect of SRPIN340 has been recently demonstrated [22]. This effect was shown to be mediated by antiangiogenic activity and required dairy SRPIN340 administrations to the tumor locally due to the low pharmacological capacity of SRPIN340. This finding indicates that novel compounds with increased drug-like properties should be searched for in future studies. In recent years, a number of anticancer agents that reached clinical use were developed through ‘target-based’ approaches, mostly involving the knowledge of structural aspects of ligand-target interactions [52]. Following this rationale, the possible interaction contacts between SRPIN340 and SRPK2 evaluated herein create a path to develop novel inhibitors with higher affinities and increased drug-like characteristics [22].

Molecular dynamics simulation analyses revealed a high stability of the SRPK2/SRPIN340 complex, reinforcing the robustness of the docking assays that were performed (Fig 5). W exposure to the polar solvent across the trajectories analyzed was readily detected and indicates that the ligand binding induced conformational changes in the kinase structure. According to this finding, further intrinsic W fluorescence assays revealed that SRPIN340 caused a slight redshift and induced W fluorescence suppression in the SRPK2 spectrum (Fig 6). These observations may be explained by the exposure of W residues to the polar solvent or even by the formation of pi-stacking interactions observed between SRPIN340 and W89 in the molecular dynamics simulation of pose A, confirming that overall conformational changes occur upon SRPIN340 binding (Fig 5B and 5C).

SRPIN340 titration in the fluorescence assays also allowed estimation of the K_D_ value for the SRPK2/SRPIN340 complex at 1.6 ± 0.3 μM. Previous studies with the yeast SRPK homologue Sky1p revealed K_D_ values for the ATP analogue and ATP equal to 650 μM and 2000 μM, respectively [53]. Considering these values, SRPIN340 binding seems to occur with higher affinity than would be expected for adenine nucleotides. Although comparisons between these data should be made carefully, the huge differences in these values might indicate that the ATP-competitive SRPIN340 indeed accesses different interacting groups at the ATP binding pocket because they were obtained using different experimental methodologies.

The possible binding modes analyzed here presented different interactions made by SRPIN340. They were classified by their distances’ standard deviations (Table 2) as good (SD < 0.5 Å), medium (0.5 Å < SD < 1.0 Å) and long (SD > 1 Å). For pose A, contacts on the benzenic ring (A108), CF_3_ (P247) and the pyridine ring (W89) groups were more stable, corroborating the hypothesis of hydrophobic forces playing a critical role in SRPIN340 binding. In addition, interactions A108 and F166 with the piperidine ring and interactions L87 and V95 with the benzenic ring created a suitable environment for SRPIN340 accommodation. Together, these interactions led to a binding mode of a type I kinase inhibitor. However, these contacts can be further improved, along with the ones with higher deviations, to design more suitable SRPK inhibitors. Although all structural analyses were performed considering SRPK2, interpretations may also be applied to SRPK1, considering the high similarity in their kinase domains [10,47] (S3 Fig). Additionally, SPHINX appears to be equivalent to SRPIN340 in terms of chemical properties and *in vitro* and *in vivo* activities [43,54], and the structural information obtained here may therefore complement the biological and biochemical data already available in the literature for both compounds. Considered together, all of this information will certainly sustain efforts to pursue next-generation SRPK inhibitors.

In conclusion, SRPKs were found overexpressed in myeloid and lymphoid leukemia. The SRPK inhibitor SRPIN340 presented a selective cytotoxic effect against leukemia cell lines. This effect was accompanied by the triggering of apoptosis, and it was due to intracellular SRPK activity impairment. Finally, the structural insights obtained should favor the design of SRPK inhibitors with increased potency and drug-like properties.

## Supporting Information

S1 Fig RT-qPCR analysis comparing SRPK2 mRNA expression in Molt4 and Nalm6 leukemia cells in relation to PBMC. mRNA expression analysis (A) shows that SRPK2 has higher expression in Molt4 and Nalm6 compared with non-transformed PBMC. Because all genes amplified to be used as endogenous controls strongly varied between the PBMC and leukemia cells (see graphs B-D), the data were normalized using the unit of mass of the starting material [55,56]. For this analysis, equal amounts of total RNA and cDNAs were carefully determined spectrophotometrically, allowing us to plot the relative expression values as 2^ΔCt^, where PBMC was used as a calibrator (ΔCt = Ct_(PBMC)_—Ct_(SRPK)_). The same approach was attempted with SRPK1, but its expression could not be precisely compared with the leukemia cells (see graph E) because it was barely detected in the PBMC samples. Nevertheless, this indicates that SRPK1 has very low expression in PBMC, which is in good agreement with our WB assays (Fig 1A) and with previous RT-qPCR reports [23,24]. The primers used in these experiments are detailed in S1 Table.
(TIF)

S2 Fig Effect of SRPIN340 treatment on MAP2K1, MAP2K2, VEGF and FAS expression in HeLa cells. RT-PCR was performed using primers specific for MAP2K1, MAP2K2, VEGF, and FAS genes, and cDNA were derived from HeLa cells after 18 h of treatment with SRPIN340 (100 μM). Cells treated with the vehicle DMSO were used as a control. One representative experiment of three is shown. (*) MAP2K1 splicing variant as previously described [19].
(TIF)

S3 Fig Superposition of SRPK1 and SRPK2 crystallographic structures. SRPK1 (PDB ID 1WAK, grey) and SRPK2 (PDB ID 2X7G, blue) structures were aligned attesting their high similarity.
(TIF)

S1 Table List of primers. (PDF)
