# Supplementary figures and images for: Potential Antileukemia Effect and Structural Analyses of SRPK Inhibition by N-(2-(Piperidin-1-yl)-5-(Trifluoromethyl)Phenyl)Isonicotinamide (SRPIN340)
Source: PLoS One. 2015 Aug 5;10(8):e0134882. doi: 10.1371/journal.pone.0134882 (PMC4526641; doi:10.1371/journal.pone.0134882)

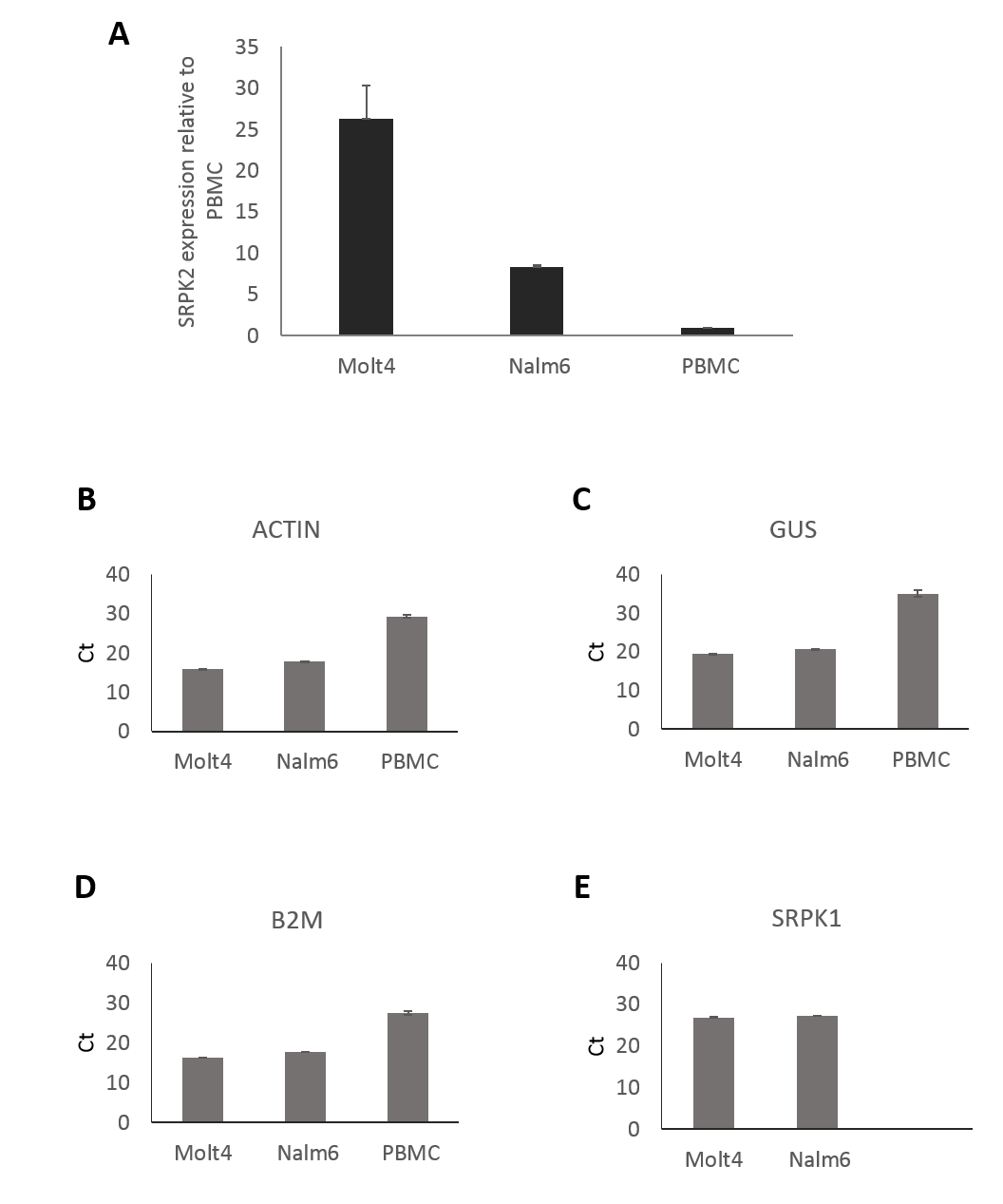

Supplement: S1 Fig — mRNA expression analysis (A) shows that SRPK2 has higher expression in Molt4 and Nalm6 compared with non-transformed PBMC. Because all genes amplified to be used as endogenous controls strongly varied between the PBMC and leukemia cells (see graphs B-D), the data were normalized using the unit of mass of the starting material [55,56]. For this analysis, equal amounts of total RNA and cDNAs were carefully determined spectrophotometrically, allowing us to plot the relative expression values as 2ΔCt, where PBMC was used as a calibrator (ΔCt = Ct(PBMC)—Ct(SRPK)). The same approach was attempted with SRPK1, but its expression could not be precisely compared with the leukemia cells (see graph E) because it was barely detected in the PBMC samples. Nevertheless, this indicates that SRPK1 has very low expression in PBMC, which is in good agreement with our WB assays (Fig 1A) and with previous RT-qPCR reports [23,24]. The primers used in these experiments are detailed in S1 Table. (TIF) [file pone.0134882.s001.tif]

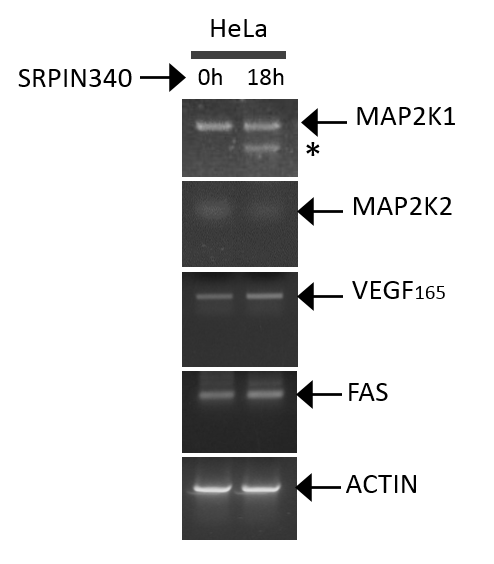

Supplement: S2 Fig — RT-PCR was performed using primers specific for MAP2K1, MAP2K2, VEGF, and FAS genes, and cDNA were derived from HeLa cells after 18 h of treatment with SRPIN340 (100 μM). Cells treated with the vehicle DMSO were used as a control. One representative experiment of three is shown. (*) MAP2K1 splicing variant as previously described [19]. (TIF) [file pone.0134882.s002.tif]

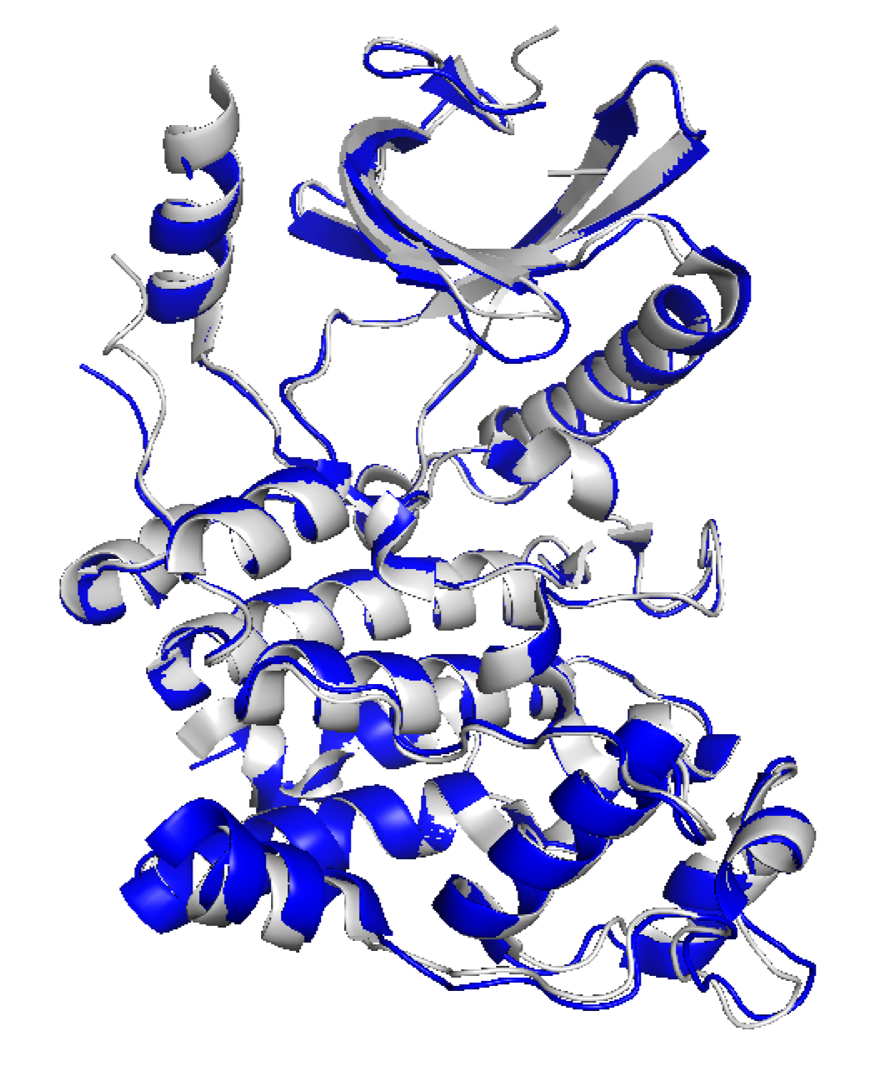

Supplement: S3 Fig — SRPK1 (PDB ID 1WAK, grey) and SRPK2 (PDB ID 2X7G, blue) structures were aligned attesting their high similarity. (TIF) [file pone.0134882.s003.tif]
