# Supplementary material for: Potential Antileukemia Effect and Structural Analyses of SRPK Inhibition by N-(2-(Piperidin-1-yl)-5-(Trifluoromethyl)Phenyl)Isonicotinamide (SRPIN340)
Source: PLoS One. 2015 Aug 5;10(8):e0134882. doi: 10.1371/journal.pone.0134882 (PMC4526641; doi:10.1371/journal.pone.0134882)
Supplement: S1 Table — (PDF) [file pone.0134882.s004.pdf]

S1 Table. List of primers.

**RT-PCR primers:**		
Primer Name	Primer sequence 5'→3'	Reference
ACTIN_F<br>ACTIN_R	CCAGCTCACCATGGATGATGATATCG<br>GGAGTTGAAGGTAGTTTCGTGGATGC	[19]
FAS_F<br>FAS_R	TCAAGGAATGCACACTCACC<br>TCCTTTCTGTGCTTTCTGCAT	[44]
MAP2K1_F<br>MAP2K1_R	CCAAAATGCCCAAGAAGAAGCCG<br>CCAAACACTTAGACGCCAGCAGC	[19]
MAP2K2_F<br>MAP2K2_R	CACCATCAACCCTACCATCGCC<br>CCACTTCTTCCACCTCGGACC	[19]
VEGF_F<br>VEGF_R	GTAAGCTTGTACAAGATCCGCAGACG<br>ATGGATCCGTATCAGTCTTTCCTGG	[32]
**Quantitative RT-PCR primers:**		
Primer Name	Primer sequence 5'→3'	Reference
ACTIN_F<br>ACTIN_R	TGGATCAGCAAGCAGGAGTATG<br>GCATTTGCGGTGGACGAT	_
GUS_F<br>GUS_R	GAAAATATGTGGTTGGAGAGCTCATT<br>CAGCACTCTCGTCGGTGACTGTTCA	_
B2M_F<br>B2M_R	GAGTATGCCTGCCGTGTG<br>CCTCCATGATGCTGCTTACATGTCTC	_
SRPK1_F<br>SRPK1_R	TGCTTGTTGGGTGCACAAA<br>CGATTAGAACTTCCAAGGAACGA	_
SRPK2_F<br>SRPK2_R	GCAAAGGACAATGGTGAAGCTGAGG<br>CATCATCATCTTCATCGTCCAGTTGC	_
